# Organized Unidirectional Waves of ATP Hydrolysis within a RecA Filament

**DOI:** 10.1371/journal.pbio.0030052

**Published:** 2005-02-08

**Authors:** Julia M Cox, Oleg V Tsodikov, Michael M Cox

**Affiliations:** **1**Department of Biochemistry, University of WisconsinMadison, WisconsinUnited States of America; Brandeis UniversityUnited States of America

## Abstract

The RecA protein forms nucleoprotein filaments on DNA, and individual monomers within the filaments hydrolyze ATP. Assembly and disassembly of filaments are both unidirectional, occurring on opposite filament ends, with disassembly requiring ATP hydrolysis. When filaments form on duplex DNA, RecA protein exhibits a functional state comparable to the state observed during active DNA strand exchange. RecA filament state was monitored with a coupled spectrophotometric assay for ATP hydrolysis, with changes fit to a mathematical model for filament disassembly. At 37 °C, monomers within the RecA-double-stranded DNA (dsDNA) filaments hydrolyze ATP with an observed *k*_cat_ of 20.8 ± 1.5 min^−1^. Under the same conditions, the rate of end-dependent filament disassembly (*k*_off_) is 123 ± 16 monomers per minute per filament end. This rate of disassembly requires a tight coupling of the ATP hydrolytic cycles of adjacent RecA monomers. The relationship of *k*_cat_ to *k*_off_ infers a filament state in which waves of ATP hydrolysis move unidirectionally through RecA filaments on dsDNA, with successive waves occurring at intervals of approximately six monomers. The waves move nearly synchronously, each one transiting from one monomer to the next every 0.5 s. The results reflect an organization of the ATPase activity that is unique in filamentous systems, and could be linked to a RecA motor function.

## Abstract 2

The RecA filament plays a key role in bacterial DNA recombination and repair. Biochemical studies now reveal some unexpected properties of the filament

## Introduction

There are three prominent protein families that both form filaments and hydrolyze nucleoside triphosphates (NTPs). These include the tubulins [1,2,3], the actins [4,5,6], and RecA protein and its homologs [7,8,9,10,11]. Of these, the RecA family is unique in its formation of filaments on a DNA cofactor and in the steady-state hydrolysis of ATP by monomers within an assembled filament [10,11,12]. The function of RecA-mediated ATP hydrolysis has been a source of conjecture and debate for over two decades [10,11,13,14,15,16,17,18,19,20,21].

The bacterial RecA protein promotes the central steps of recombinational DNA repair [12,22]. In vitro, the RecA protein catalyzes a DNA strand exchange reaction that mimics the presumed function of RecA protein in vivo. The RecA protein first forms a filament on single-stranded DNA (ssDNA). The bound ssDNA is then aligned and paired with a homologous double-stranded DNA (dsDNA), forming a short (up to approximately 1 kilobasepair [kbp]) segment of paired DNA and initiating the DNA strand exchange [11,12]. The initial paired DNA can be extended in a reaction that generally requires ATP hydrolysis [10,11,12].

**Figure 1:**
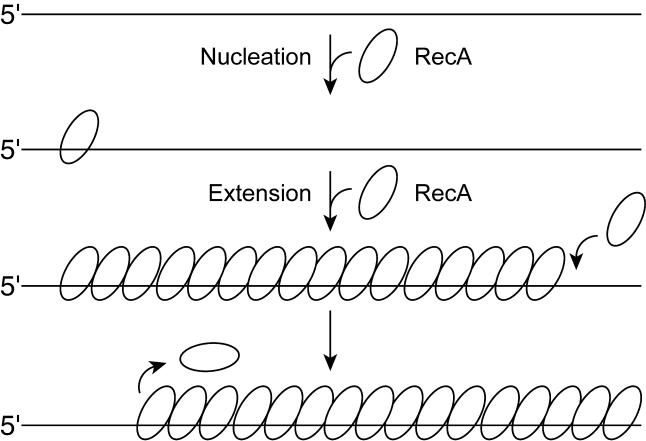
RecA Filament Assembly and Disassembly on ssDNA The reaction is limited by a slow nucleation step, followed by rapid extension in the 5′ to 3′ direction. Disassembly is also uniquely 5′ to 3′, proceeding from the end opposite to that where extension occurs. Dissociation of RecA monomers at the disassembling end requires ATP hydrolysis (see text).

RecA filaments are assembled and disassembled in an end-dependent fashion. Filament assembly proceeds in steps, with a slow nucleation followed by a very rapid 5′ to 3′ extension phase to coat the available DNA [11,12,23,24,25]. The rapid extension phase does not limit overall filament assembly within the pH range normally used for RecA experiments [26,27]. Filament disassembly is also end-dependent and proceeds 5′ to 3′ such that monomers are subtracted from filaments at the end opposite to the end where monomers are added in the extension process (Figure 1) [25,28,29]. In studies carried out to date, we have not detected RecA monomer addition to the disassembling end or RecA monomer subtraction from the assembly (extension) end [25,27,28], although both processes presumably occur at some low rate.

The RecA protein also binds to dsDNA, but nucleation onto dsDNA is much slower than onto ssDNA [26,30]. Nucleation directly onto dsDNA is pH-dependent, occurring more rapidly as the pH declines from 7.0 to 6.0 [30,31]. At any pH, filament extension on dsDNA is rapid, with complete filaments incorporating thousands of RecA monomers within a few minutes [25,32,33,34]. Upon binding dsDNA, RecA underwinds the DNA by approximately 40% and extends the helix to about 18 basepairs (bp) per turn relative to the B-form helix [35,36]. A RecA monomer binds three nucleotides (nt) of ssDNA or 3 bp of dsDNA, so one helical turn of the nucleoprotein filament includes approximately six RecA monomers.

RecA protein within a filament hydrolyzes ATP in a reaction that is almost completely DNA-dependent under standard reaction conditions [31]. Under our normal reaction conditions, the intrinsic *k*_cat_ values for ssDNA and dsDNA-dependent ATP hydrolysis rates are approximately 30 and 20 min^−1^, respectively, as determined in multiple trials over the past two decades [10,11,12]. ATPase rates are independent of pH in the range 6–9 [30,31]. ATP hydrolysis occurs uniformly throughout the filament of RecA-DNA complexes, and there is no detectable change or enhancement at filament ends [37]. In the presence of ATP analogs that are not hydrolyzed, RecA protein will form filaments on DNA and promote substantial DNA pairing and strand exchange [38,39,40]. However, RecA must hydrolyze ATP to bring about net filament disassembly [25,27,28,29,32,33,34], bypass of heterologous inserts during DNA strand exchange [41,42], DNA strand exchange with DNA substrates greater than 3 kbp in length [43], and DNA strand exchange between two duplex DNA molecules [42,44,45]. It is not clear how RecA protein-mediated ATP hydrolysis is coupled to these functions. Intriguingly, the core domain of the RecA protein (residues 34–269) is structurally homologous to several motor proteins, including hexameric helicases [46] and the mitochondrial F_1_-ATPase [47]. Models for a motor-like coupling of ATP hydrolysis to DNA strand exchange in certain DNA metabolic situations have been proposed [10].

**Figure 2:**
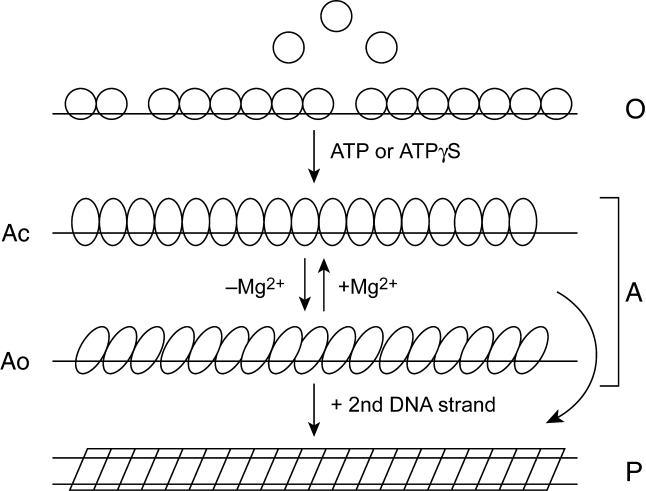
Four Structural States of RecA Protein The O state is present in the absence of ATP or ATP analogs, whether the protein is free in solution or bound to DNA. The A state is found on ssDNA in the presence of ATP. Two versions of the A state, Ac and Ao, are found at different Mg^2+^ concentrations. Addition of a second DNA strand to the RecA filament converts it to the P state.

There is now evidence for at least four different functional states of RecA protein, occurring at different reaction stages. These have recently been designated O, Ac, Ao, and P [10,48,49] (Figure 2). The O state is largely inactive and found in the absence of nucleotide cofactors or in the presence of ADP [50]. RecA in the O state can bind to DNA, creating a helical filament with a pitch of 76 Å [50]. Addition of ATP, ATPγS, or dATP results in a conformation change to an active form that is manifested by extended filaments on DNA with a pitch of 95 Å [50,51,52,53,54,55,56,57,58]. When RecA filaments form on ssDNA, they are in a structural and functional state designated A. Interconversion between the two different A states, which have somewhat different properties, is mediated largely by the Mg^2+^ concentration. However, all RecA filaments on ssDNA hydrolyze ATP with a *k*_cat_ of approximately 30 min^−1^. Addition of a second DNA strand to a RecA filament, as in a filament bound to dsDNA or a filament promoting DNA strand exchange, converts the filament to the P state. The P state is characterized by 30% lower rates of ATP hydrolysis [59,60], higher rates of exchange of RecA monomers into and out of the filament [61,62], and a higher degree of cooperativity in the ATPase function [27,49,61] than the A conformations.

How can RecA monomers be added to one end of a filament and deleted from the other end in the same test tube? The monomer-monomer interfaces are presumably the same at either end (and everywhere else) in the filament. As pointed out by Wegner [63], the dissociation constant, *K*_D_, for monomer addition to either filament end cannot be different unless an independent source of chemical energy is provided to affect the binding to one end or the other. As noted above, filament assembly does not require ATP hydrolysis, but filament disassembly does [25,27,28,29,32,33,34]. The hydrolysis of ATP by interior monomers does not generally result in dissociation, and under some conditions ATP hydrolysis can proceed with no evident dissociation of RecA monomers [25,61,64]. A simple model arises: ATP hydrolysis occurs everywhere, resulting in dissociation only for monomers at a disassembling end.

Within a RecA filament, ATP hydrolysis could occur at random, or it could be highly organized as cooperative waves traveling through the filament. There is no convenient method for monitoring the effects of ATP hydrolysis in the middle of a RecA filament. However, important clues can be ascertained by examining one effect of ATP hydrolysis: the 5′ to 3′ end-dependent filament disassembly process. In effect, we cannot see waves of ATP hydrolysis within a filament, but we can monitor one of the waves—the one occurring at the disassembling filament end.

The rate of disassembly can lead directly to an assessment of the degree of coupling of the ATP hydrolytic cycles of adjacent RecA monomers within the filament. For example, since RecA hydrolyzes ATP at a rate of approximately 30 min^−1^ on ssDNA, each RecA monomer is hydrolyzing one ATP molecule every 2 s. If the ATP hydrolytic cycles of adjacent monomers are not coupled in any way, this will be reflected in a predictable rate of filament disassembly. When the end monomer hydrolyzes ATP and dissociates, the next monomer in line could be at any point within its ATP hydrolytic cycle. However, in a large population of filaments, the next monomer will be on average halfway through the cycle, and it will hydrolyze ATP (and dissociate) one second later. Dissociation of one RecA monomer per second will lead to a measured disassembly rate of 60 monomers of RecA per minute per filament end. This is close to the situation observed for RecA monomers formed on ssDNA (functional state A), which disassemble with a rate of 60–70 monomers per minute per filament end [27]. Note that this is the slowest rate of disassembly compatible with the rate of ATP hydrolysis observed under the conditions of this experiment, and requires that every ATP hydrolytic event occurring in the RecA monomer at the disassembly end of the filament results in dissociation of that monomer. If the probability of dissociation upon ATP hydrolysis is less than 100%, the disassembly process would have to be slower than is observed.

**Figure 3:**
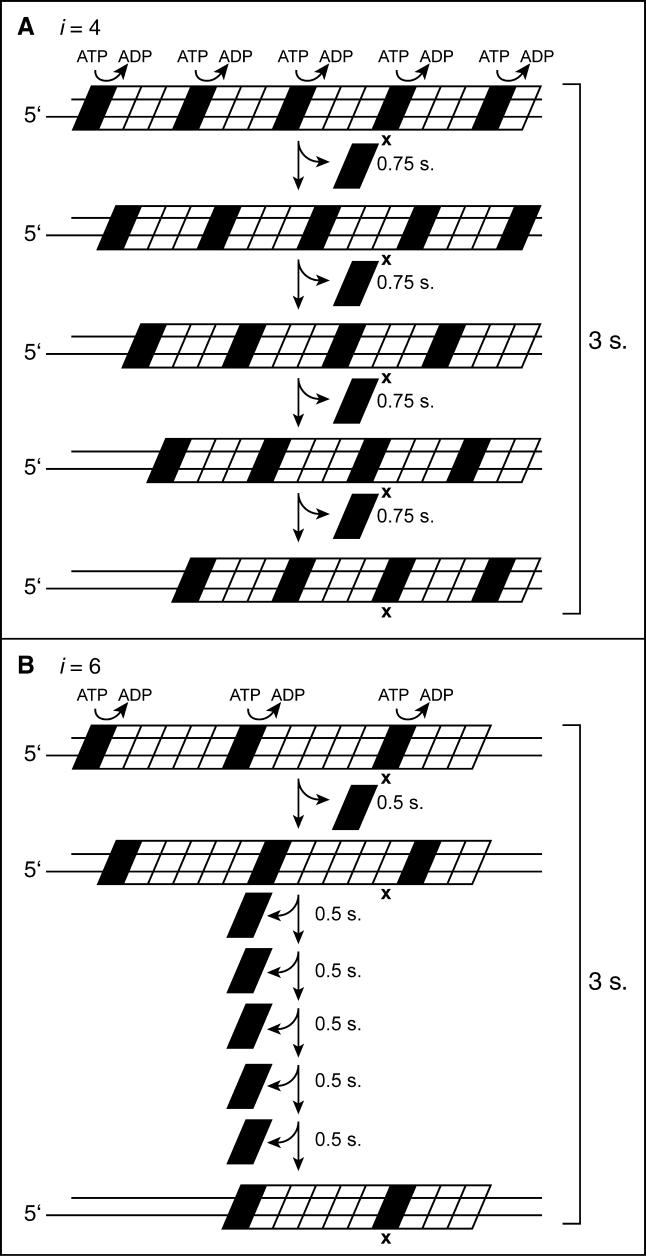
Coupled Waves of ATP Hydrolysis in RecA Filaments (A) The interval between hydrolyzing monomers *(i)* is set at four monomers. The dark monomers are those at the hydrolytic step of their ATP hydrolytic cycle. Each monomer within the filament (e.g., the one marked with an “X”) is hydrolyzing an ATP every 3 s, so that a new wave must reach it within that time span. If *i* = 4 monomers, the waves must move every 0.75 s. The last wave, at the disassembling end, results in dissociation. (B) The same considerations in (A) for *i* = 4 monomers are illustrated for *i* = 6 monomers.

A coupling of the ATP hydrolytic cycles of adjacent monomers could in principle lead to greater rates of filament disassembly. We are particularly interested in the status of RecA filaments formed on dsDNA and those promoting DNA strand exchange. As indicated above, these filaments are in the P functional state and exhibit a higher degree of coupling involving the hydrolytic cycles of adjacent monomers than is seen for filaments formed on ssDNA. If ATP hydrolysis is organized into cooperative waves traveling through the filament, then the rate of disassembly from dsDNA will reveal the rate of movement of one of the waves and the interval, *i,* between successive waves within the entire filament. For example, consider monomers within RecA filaments on dsDNA that are hydrolyzing ATP at approximately 20 min^−1^ (or one ATP every 3 s). If ATP hydrolytic cycles of adjacent monomers are coupled within the filament so that ATP hydrolysis is organized in waves (Figure 3), then a new wave must reach a given monomer every 3 s to account for the observed *k*_cat_. If the waves are four monomers apart (*i* = 4 monomers, Figure 3A), a wave must move from one monomer to the next one in line every 0.75 s. At the disassembling end (the ultimate wave), one monomer would dissociate every 0.75 s, giving a disassembly rate of 80 monomers min^−1^ filament end^−1^. If instead the waves are organized at intervals of six monomers (Figure 3B), then to reach a given monomer every 3 s, a wave would have to move from one monomer to the next every 0.5 s. At the disassembling end, a monomer would dissociate every 0.5 s to yield a disassembly rate of 120 monomers min^−1^ filament end^−1^. We define the rate of end-dependent filament disassembly as *k*_off_. Note that the numerical relationship between *k*_off_ and the *k*_cat_ for ATP hydrolysis by individual monomers reveals the distance between waves within the filament, *i*, such that *k*_off_/*k*_cat_ = *i*. For the examples above, 80/20 = 4, and 120/20 = 6.

The *k*_cat_ for ATP hydrolysis for RecA monomers on dsDNA is readily measured. An accurate determination of *k*_off_, as needed to determine *i,* is a more substantial challenge that is met in this report.

## Results

### Experimental Design

**Figure 4:**
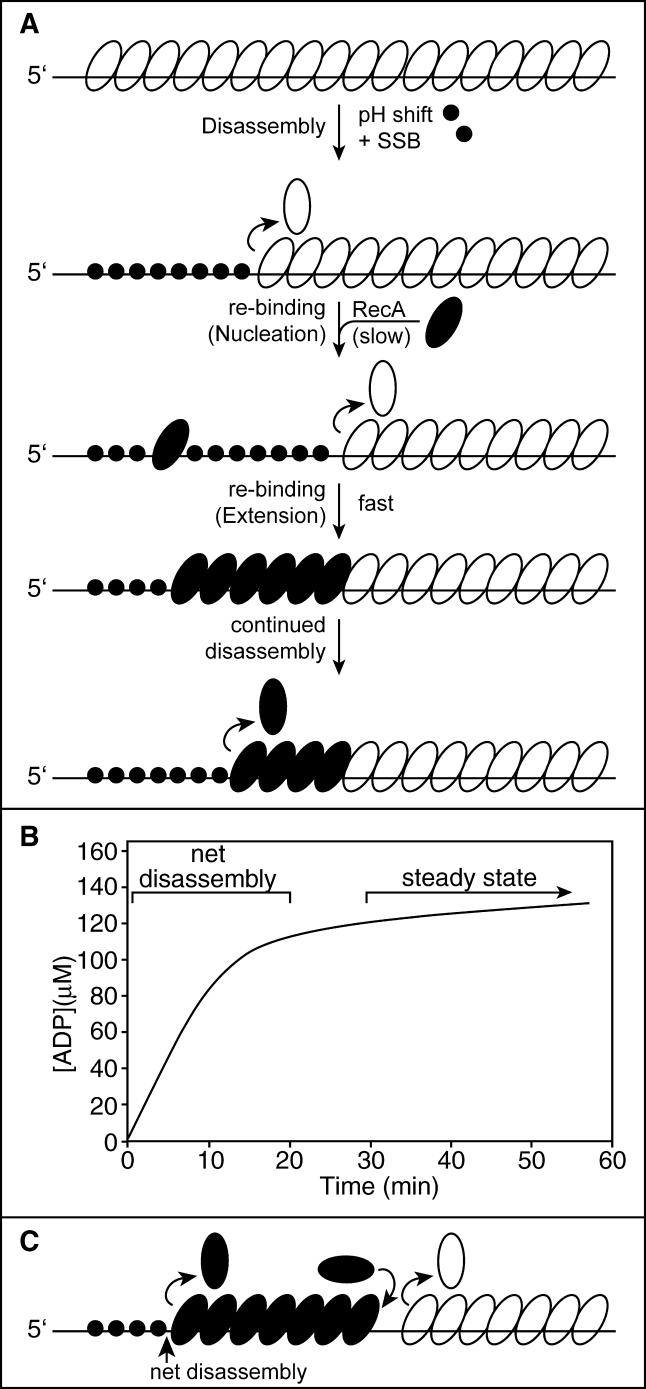
Model for RecA Filament Disassembly from Linear ssDNA (A) In the model, end-dependent disassembly occurs, with SSB filling in the vacated DNA. At low frequency, additional RecA monomers nucleate filament formation on the vacated DNA, and the new filament (dark ovals) extends until it catches up with the original filament (open ovals). This creates a new disassembling end. (B) Kinetics of disassembly as monitored by DNA-dependent ATP hydrolysis. This curve is an approximation of curves reported in previous work [27]. The rate of ATP hydrolysis declines as RecA protein dissociates from the ssDNA, until a lower steady-state rate is reached. The steady state reflects a balance between disassembly and new filament formation. (C) If, upon rebinding, the new filament does match the old one in phase, the junction between the old and new filaments could be a site of RecA monomer exchange with the solution. There will be no net disassembly at this point and no resulting change in the rate of ATP hydrolysis, because any monomers that dissociate will be immediately replaced by extension of the trailing filament.

A model system has been developed that allows the rate of end-dependent RecA filament disassembly from ssDNA to be determined quantitatively by monitoring the rate of ATP hydrolysis during the disassembly process [10,27]. The system is outlined in Figure 4A. RecA filaments are assembled on a linear ssDNA at a low pH, at which nucleation is fast enough that the DNA is saturated with RecA and there is little net disassembly (disassembling RecA monomers are rapidly replaced). The reaction mixture is then shifted to a higher pH, at which the rate of filament nucleation is slower and net filament disassembly can be observed. The rate of ATP hydrolysis will decline as RecA monomers dissociate from the DNA [27], reaching a nonzero steady-state endpoint (Figure 4B). The steady-state rate reflects the final balance between disassembly and renucleation of RecA protein and filament formation on the ssDNA vacated by the disassembling filament. Since filament extension is very fast relative to disassembly, any nucleation event will result in a filament that extends from the nucleation point to the disassembling end of the first filament, and in effect create a new point for disassembly (Figure 4A). On any of these DNA molecules, there is only one point where net disassembly occurs. Even when the second filament is not perfectly in phase with the first filament, such that some disassembly continues at the junction (Figure 4C), the RecA that dissociates from filament 1 at the junction is rapidly replaced by extension of filament 2 immediately behind it (filament extension is much faster than disassembly). Thus, there is no net change in bound RecA that would be reflected in the observed rates of ATP hydrolysis, except at the end of filament 2. To minimize the rebinding of RecA protein to vacated DNA, ssDNA-binding protein of Escherichia coli (SSB protein) is added at the time of the pH shift [27]. Bound SSB limits the nucleation of RecA filaments on ssDNA [25,65,66,67]. This in turn permits a measurable decline in ATP hydrolysis.

The process in Figure 4 is modeled by equation 1, where *k*_nuc_ is the rate of renucleation of RecA filaments to vacated DNA during the disassembly process, *k*_off_ is the end-dependent disassembly rate as noted in the Introduction, *n*_tot_ is the total number of RecA protein binding sites on the DNA molecule used as substrate, [D-ends] is the concentration of disassembling ends, and *k*_cat_ is the turnover number for ATP hydrolysis by RecA monomers in the filament.



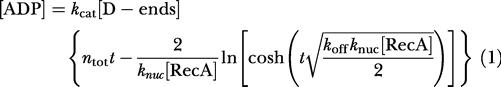



The derivation of equation 1 is described in detail elsewhere [27]. The assumptions incorporated into the equation and the model of Figure 4 are also detailed elsewhere [27]. In brief, the assumptions are as follows. First, nucleation of filament formation is the rate-limiting step for RecA filament assembly. This assumption is documented in the Introduction of this paper and in previous reports [23,27]. Second, filament extension is faster than filament disassembly under all conditions. The simplest of the arguments [27] underpinning this assertion is that filaments would never form on DNA if RecA were subtracted from the disassembling end faster than it could be added to the extending end. Third, the model assumes that net disassembly is occurring at only one point in each filament. This is addressed in Figure 4C and follows from the fact that filament extension is faster than filament disassembly. There will be no net disassembly except at the 5′ proximal end of the RecA protein tracts on a given linear DNA. Any dissociation in the middle of a filament is rapidly replaced by the growth of trailing filament segments and thus cannot contribute to a net change in ATP hydrolysis. Finally, the assumption is made that the *k*_cat_ for RecA-mediated ATP hydrolysis does not change in the range used for the pH shifts. This is documented elsewhere [26,27,30].

**Figure 5:**
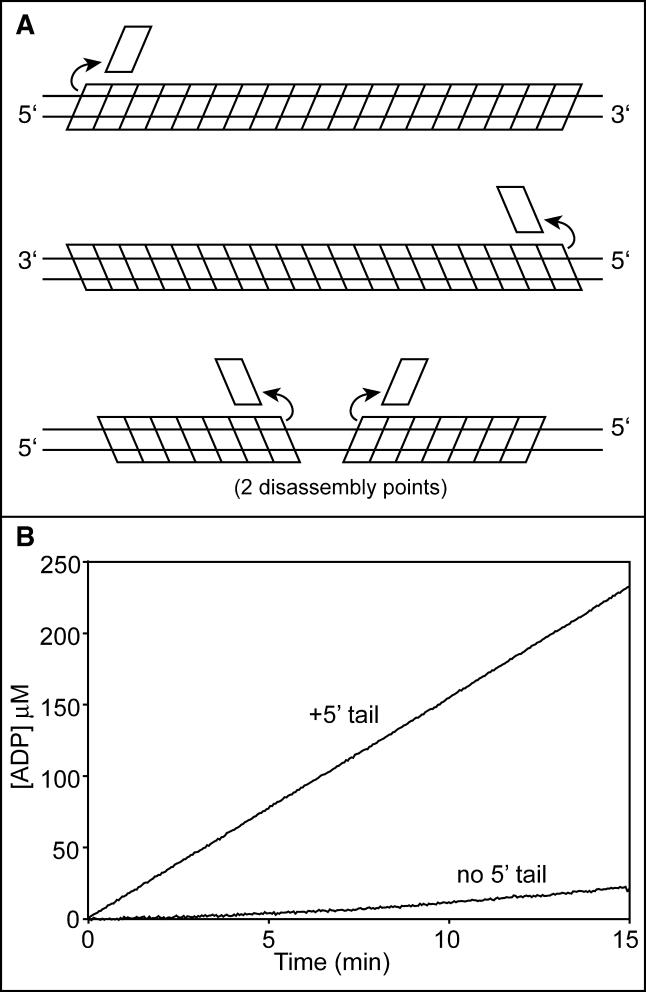
RecA Filament Formation on dsDNA (A) RecA filaments can nucleate on dsDNA so as to have two different orientations. The initiating strand guides the orientation of each filament, with its polarity labeled in each panel. If two nucleations occur on the same filament, there can be two independent disassembly points. (B) The effect of the 30-nt 5′ extensions on RecA protein filament formation. Reactions contained 4 μM RecA protein, 4 μM DNA, and no SSB and were carried out at pH 6.6. The tailed DNA substrate has 2,900 bp of duplex DNA with the 30-nt 5′ extension on one end. The completely duplex DNA is 3,162 nt in length. The enhanced nucleation afforded by these tails is evident in the rapid ATP hydrolysis seen when the tailed DNA is used as cofactor.

On dsDNA, the situation becomes more complicated because it is more difficult to define the orientation of the filaments. RecA protein binds to the two strands of a duplex DNA unequally. One strand is bound at the site normally occupied by ssDNA. This strand orients the filament, and it is protected from nuclease digestion better than the second strand of a duplex [33,68]. We will call this first strand the initiating strand [22]. In principle, either strand of a duplex can act as the initiating strand and organize the 5′ to 3′ assembly of RecA filaments. RecA filaments can thus form in two orientations on dsDNA (Figure 5A). If nucleation occurs in the middle of a linear duplex so that the filament extends to one end, and a second nucleation occurs on the same DNA but in the opposite orientation, there could be two filaments on the same DNA, both with disassembling ends (Figure 5A). This in turn would complicate the mathematical modeling of the disassembly reaction. This potential problem can be alleviated by supplying a good nucleation site in the form of a stretch of ssDNA. At pH 8 and above, RecA nucleation onto duplex DNA becomes very slow (measured in hours) [26,30]. If a 5′ single-strand extension is added to one end of the DNA, the nucleation occurs within a few minutes, and RecA readily extends from the single-stranded segment into the adjoining duplex [33]. A 3′ single-strand extension does not work [33], as would be predicted from the polarity of filament assembly from a nucleation site. This effect is seen in the experimental data presented in Figure 5B. RecA binds to a linear duplex of 3,162 bp only after a lag lasting tens of minutes, even at pH 6.6. A linear duplex of similar length, but this time with a 5′ single-strand extension of 30 nt, is bound without a measurable lag at the same pH. This indicates that the filaments are nucleating on the single-strand extension, and virtually all of the resulting filaments in the population will have a unique orientation over the entire length of the DNA that is dictated by the single-stranded tail.

**Figure 6:**
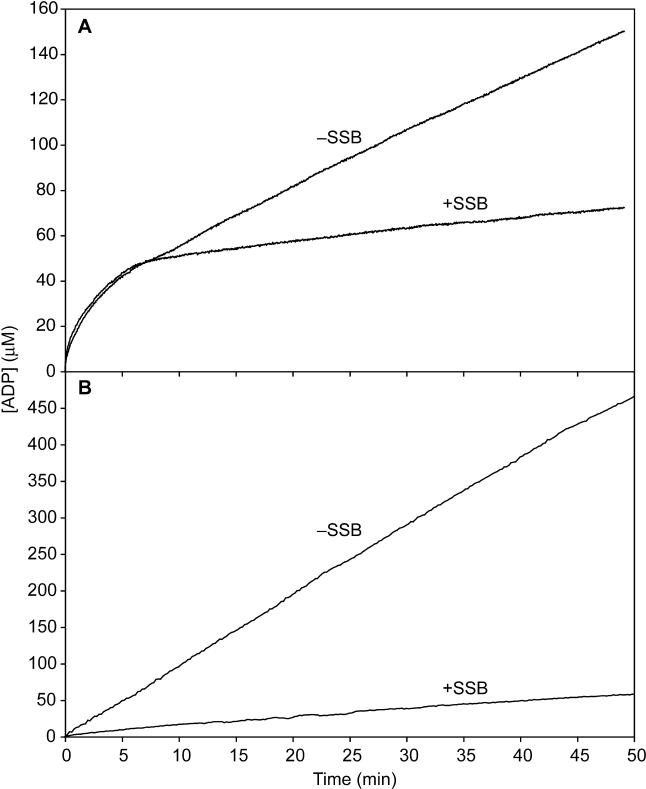
The Effect of SSB Protein in Suppressing Rebinding of RecA to the Single-Strand Tails (A) Normal RecA filament disassembly protocol. The RecA filaments were formed on the tailed DNA at pH 6.62. The pH was then shifted to 8.0 to allow disassembly to commence. Disassembly curves are shown in the presence and absence of SSB. Reactions contained (after the pH shift) 2 μM RecA protein, 2 μM DNA, and (where indicated) 0.05 μM SSB. (B) Direct binding of RecA protein to the tailed DNA at pH 8.0. Reactions contained 6 μM RecA protein, 6 μM DNA, and (where indicated) 0.15 μM SSB. When included, SSB was added prior to the RecA protein.

To isolate the disassembly process so that it can be measured accurately, we must limit the rate of RecA rebinding to DNA that has been vacated as the RecA dissociates. Several parameters can be adjusted to accomplish this. The first one is pH. The rate of RecA protein disassembly reaches an apparent maximum at pH 8 and above [33]. The rate of disassembly should reach a maximum when every ATP hydrolytic event at the disassembling end results in dissociation of a RecA monomer. The rate of nucleation on dsDNA declines with increased pH such that working at high pH minimizes RecA rebinding. The second parameter is DNA length, which can affect the consequences of rebinding to duplex DNA. A nucleated filament is rapidly extended to the DNA end. A nucleation event on a long DNA will generate more bound RecA than a nucleation event on a short DNA. Thus, the use of shorter DNAs will limit the effects of each nucleation event. However, the DNA length cannot be reduced too much to suppress the levels of RecA protein rebinding, because a longer DNA (and filament) allows for a more gradual decline in the ATPase reaction that is easier to monitor. Trials using DNAs of several lengths indicated that a linear dsDNA with a length of approximately 3 kbp was optimal for this work [33,69]. These dsDNAs result in bound RecA filaments long enough (about 1,000 monomers) to disassemble over a time period of multiple minutes, while limiting the effects of RecA rebinding. As shown later, we also carried out measurements using dsDNAs of 2 kbp and 4 kbp. A third parameter that can be adjusted is SSB concentration. Adding SSB when the pH shift is carried out inhibits the rebinding of free RecA protein to the ssDNA extension. The concentrations of SSB used in these experiments were determined empirically to provide the maximum possible suppression of RecA renucleation. The effectiveness of this SSB-mediated suppression is illustrated in Figure 6.

**Figure 7:**
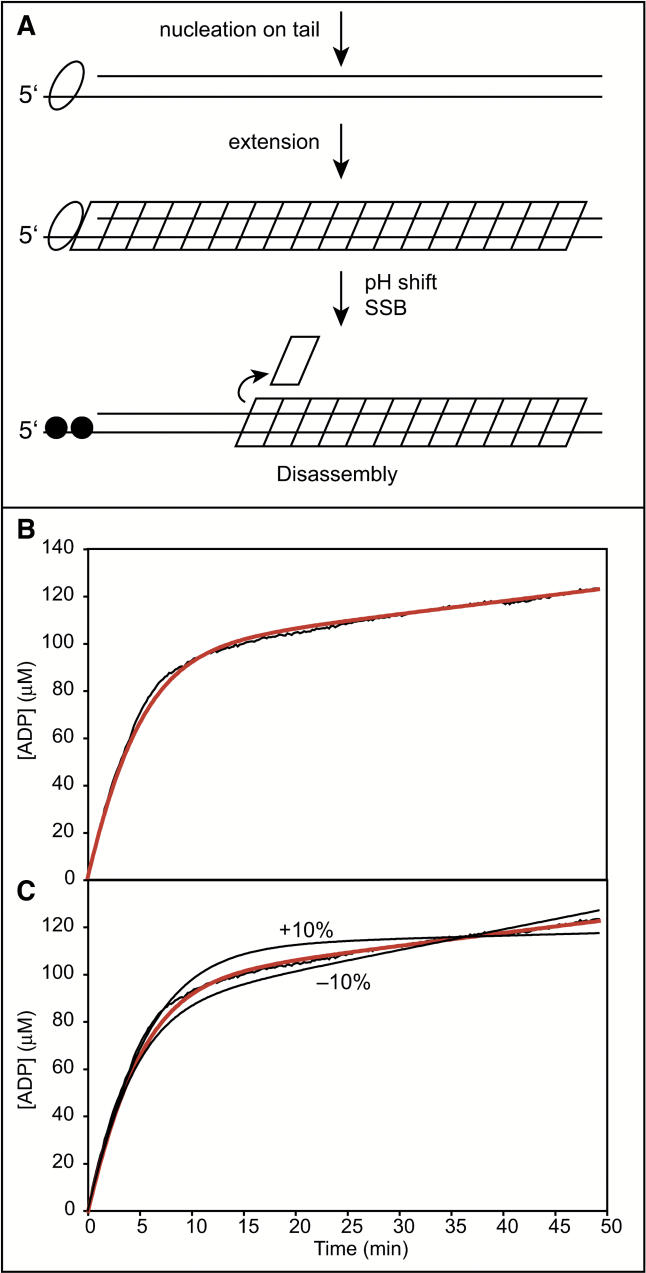
Quantitative Measurement of RecA Filament Disassembly from dsDNA (A) The experimental protocol, as described in the text. (B) A typical disassembly curve. The black line is the measured ADP production curve. The orange line is the fitting of the data by equation 1. (C) The curve is identical to that shown in panel B for the 3-kbp DNA substrate, with the best fit line shown in orange. However, two additional curves (in solid black) are shown to illustrate the variation in fitting if the value of *k*_off_ is constrained to values 10% above or 10% below the best-fit determination.

Optimizing all of these considerations, the general protocol for these experiments is outlined in Figure 7A. A linear duplex of 2,900 bp, with a 30-nucleotide 5′ single-strand extension, is bound with RecA protein at pH 6.62. The DNA concentration is 12 μM, so that the concentration of RecA binding sites is 2 μM. The RecA protein is added in excess (12 μM) to facilitate saturation of the DNA. After binding is complete, the rate of ATP hydrolysis is measured. The pH is then shifted abruptly to 8 by diluting the solution 2-fold into a solution with another buffer. The RecA and DNA concentrations undergo a 2-fold decrease during the pH shift, but the concentration of ATP and ATP regeneration components are kept constant. The pH shift initiates a net disassembly process that leads to a decline in ATP hydrolysis. Rebinding of RecA protein to the vacated single-strand extension is suppressed by SSB added with the pH shift buffers. The production of ADP is monitored. The ATP regeneration system is set up so as not to limit the observed results. For example, results were the same if the phosphoenolpyruvate (PEP) concentration was reduced from 3 to 1.5 mM. The starting *k*_cat_ for ATP hydrolysis is reported as the measured rate of ATP hydrolysis prior to the pH shift (in μM min^−1^), divided by the initial concentration of bound RecA protein (in 2 μM).

With the filaments uniquely oriented on the tailed DNA and encompassing the entire length of the DNA, equation 1 can be applied to the measurement of disassembly from dsDNA in the same manner as it was used to analyze the disassembly from ssDNA. The rate of filament disassembly, *k*_off_, is obtained by fitting the equation to the ADP production curve. There is just one caveat. The renucleation onto the vacated DNA must be suppressed sufficiently so that there is, to a reasonable approximation, no more than one disassembling RecA filament end per bound DNA. If multiple filaments formed on the vacated dsDNA as a result of renucleation, this process would not be properly modeled by equation 1 (which was derived for a model in which all filaments had a single orientation [27]). As described below, the rates of renucleation are suppressed sufficiently in these experiments so that this condition is met.

### Measurement of Disassembly Rates from Duplex DNA

A typical experiment is illustrated in Figure 7B. Equation 1 relates the production of ADP in terms of the two primary unknowns, *k*_off_ and *k*_nuc_, as well as several known parameters. All concentrations are in μM, and all times are in minutes. The data from ATP hydrolysis assays were converted to [ADP], plotted as a function of time, and fit to equation 1 using the program SigmaPlot from SPSS (Chicago, Illinois, United States). The terms [D-ends], *n*_tot_, and [RecA] were known for each experiment and held constant for fitting. The terms *k*_off_ and *k*_nuc_ were the fitting parameters. The apparent *k*_cat_ for ATP hydrolysis was determined independently as the rate of ATP hydrolysis before the pH shift and initiation of net filament disassembly divided by the concentration of bound RecA protein. However, *k*_cat_ can be determined as a fitting parameter as well. Therefore, each dataset was fit to equation 1 twice: once with *k*_cat_ held constant at the value measured prior to the pH shift, and once with *k*_cat_ as a fitting parameter in addition to *k*_nuc_ and *k*_off_. The fit parameters for both fittings were compared. Experiments were discarded in which the measured and fit *k*_cat_ did not agree to within 40%, or the measured *k*_cat_ was less than 18 min^−1^ (generally corresponding to a problem with one or more reagents or equipment on that day). For the 3 kbp substrate used in Figure 7, data were collected in 16 trials carried out over a period of 3 mo. During that time, 12 trials were utilized and four were discarded on the basis of these considerations.

The plot in Figure 7B is nonlinear, reflecting the decline in the rate of ATP hydrolysis as the RecA filament disassembles from the DNA. The form of the curve is the same as that shown in Figure 4B, although the time scale is abbreviated. The rate of ATP hydrolysis settled to a slow steady-state rate in which disassembly and rebinding are balanced. The twelve trials that met the conditions elaborated above are detailed in Table 1. The result presented in Figure 7B is representative. The fitting performed with the *k*_cat_ fixed at the average value measured prior to the pH shift, *k*_cat_ = 19.5 ± 0.6 min^−1^, yields the best-fit values (averaged over 12 experiments) of *k*_off_ = 120 ± 20 min^−1^ and *k*_nuc_ = 4.6 ± 0.75 × 10^−5^ μM^−1^ min^−1^. When *k*_cat_ was allowed to vary as a fitting parameter along with *k*_off_ and *k*_nuc_, the parameters were again obtained from the best-fit curves and averaged. This yielded *k*_cat_ = 21.6 ± 4.4 , *k*_off_ = 133 ± 24 min^−1^, and *k*_nuc_ = 5.1 ± 0.89 × 10^−5^ μM^−1^ min^−1^ for the 12 datasets. The reasonable agreement between the measured and fit *k*_cat_ values in most trials helped provide confidence that the *k*_cat_ measured prior to the pH shift was consistent with the observed reaction progress curve obtained after the pH shift.

**Table 1:**
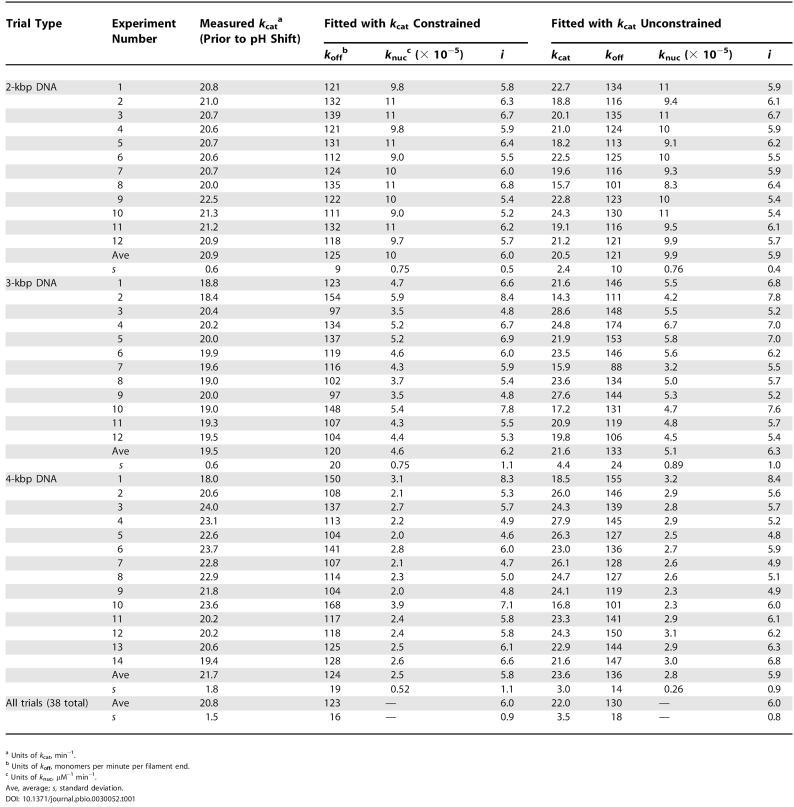
Experimental Parameters Obtained in Individual RecA Filament Disassembly Trials

^a^ Units of *k*_cat_, min^−1^

^b^ Units of *k*_off_, monomers per minute per filament end

^c^ Units of *k*_nuc_, μM^−1^ min^−1^

Ave, average; *s,* standard deviation

The fitting process is quite sensitive to small changes in the fitting parameters. Figure 7C illustrates the result of constraining *k*_off_ to values 10% above and 10% below the value obtained by fitting the data in Figure 7B for the 3 kbp DNA substrate. In both cases, the curves deviate substantially from the data and demonstrate the sensitivity of our model.

As long as the stated constraints on the use of equation 1 are met, the results should be independent of DNA length. To confirm, disassembly rates were obtained using DNA substrates of 1,961 and 3,900 bp (referred to as our 2 kbp and 4 kbp substrates, respectively). Each of these had a 5′ extension, with the same length (30 nt) and sequence as the one used for the 3-kbp DNA substrate. In this series of experiments, there were 16 trials with each DNA substrate. Of these, 12 and 14 trials (for the 2- and 4-kbp substrates, respectively) met the conditions established above for the 3-kbp DNA substrate trials, and these are reported in Table 1. The total concentration of DNA base pairs was kept constant in these experiments, so that the concentrations of DNA molecules and thus disassembling ends either increased (2-kbp substrate) or decreased (4-kbp substrate). These new trials were carried out over the course of one month.

The results agreed very well with those obtained with the 3-kbp DNA, with the *k*_cat_ and *k*_off_ values remaining constant (within experimental error) as a function of DNA length as summarized in Table 1. For the 2-kbp DNA, constraining *k*_cat_ to the value measured before the pH shift (average 20.9 ± 0.6 min^−1^) yields the best-fit values of k_off_ = 125 ± 9 min^−1^ and *k*_nuc_ = 1.0 ± 0.075 × 10^−4^ μM^−1^ min^−1^. In the same manner, the 4-kbp DNA substrate yielded average values of *k*_off_ = 124 ± 19 min^−1^ and *k*_nuc_ = 2.5 ± 0.52 × 10^−5^ μM^−1^ min^−1^ with a measured *k*_cat_ = 21.7 ± 1.8 min^−1^. When *k*_cat_ was allowed to vary as a fitting parameter along with *k*_off_ and *k*_nuc_, the best-fit values for the 2-kbp DNA substrate averaged *k*_cat_ = 20.5 ± 2.4 min^−1^, *k*_off_ = 121 ± 10 min^−1^, and *k*_nuc_ = 9.9 ± 0.76 × 10^−5^ μM^−1^ min^−1^ for the 12 datasets. The 4-kbp DNA substrate yielded best-fit values of *k*_cat_ = 23.6 ± 3.0 min^−1^, *k*_off_ = 136 ± 14 min^−1^, and *k*_nuc_ = 2.8 ± 0.26 × 10^−5^ μM^−1^ min^−1^ for the 14 datasets. All of this work is summarized in Table 1. If all of the data in Table 1 are averaged (38 total trials), the values obtained when constraining *k*_cat_ to the value measured before the pH shift are *k*_cat_ = 20.8 ± 1.5 min^−1^ and *k*_off_ = 123 ± 16 min^−1^. When *k*_cat_ was not constrained, the values were *k*_cat_ = 22 ± 3.5 min^−1^ and *k*_off_ = 130 ± 18 min^−1^. Unlike the other parameters, the nucleation of filament formation as expressed in *k*_nuc_ should be affected by factors such as G:C content and sequence structure in the duplex DNA [26,30], and was not averaged over the three sets of trials. Within a single set of trials, *k*_nuc_ was quite reproducible.

**Figure 8:**
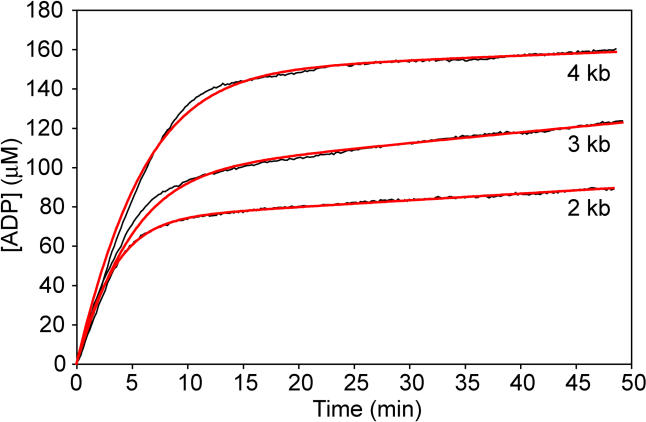
RecA Filament Disassembly Rates Are Independent of DNA Length Typical disassembly curves are shown for the 2-, 3-, and 4-kbp DNA substrates. The black lines are the data, and the orange lines represent the best fits to the data by equation 1. Note that the early, rapid phase of the curves are extended in concert with the increase in the length of the DNA substrates. The rates of disassembly derived from the curves are identical within experimental error, as summarized in Table 1 and the text.

Representative trials using the 2-, 3-, and 4-kbp DNA substrates are compared in Figure 8. The initial rates are essentially the same in each case, since the concentration of bound RecA is the same. If RecA dissociates from one end of the filament at a constant rate, disassembly of the longer filaments formed on longer DNAs should require correspondingly more time. The pattern seen here is consistent with that expectation. The final steady state rate of ATP hydrolysis reflects both *k*_nuc_ and the length of the DNA, and thus varies somewhat.

As already noted, equation 1 is useful only if there is no more than one disassembling end on a particular DNA molecule. The final steady state achieved after the major phase of filament disassembly is complete allows us to estimate the likelihood that multiple filaments with different orientations rebind to the same stretch of vacated DNA. We address this issue for the 3-kbp DNA substrate. Immediately following the pH shift, the concentration of RecA binding sites on the 6 μM DNA is 1 μM, and this represents the concentration of bound RecA at the outset. The average rate of ATP hydrolysis when disassembly was initiated was 19.5 ± 0.6 μM^−1^ min^−1.^ The average final steady-state rate of ATP hydrolysis is 1.23 μM^−1^ min^−1^, or 6.3% of the rate observed prior to disassembly. This final rate corresponds to 6.3% occupancy of the available RecA binding sites, or 0.063 μM bound RecA protein. We estimate that the average filament at steady state occupies one-quarter of the available RecA binding sites in the DNA to which it is bound from the following considerations. When a new RecA filament nucleates onto the vacated DNA, the nucleation could occur anywhere along the length of the DNA. On average, the nucleation would occur at the center of the DNA, and the fast unidirectional filament extension phase would quickly coat the DNA from that point to one end, so that the typical new filament would occupy half of the available RecA binding sites on the DNA to which it was bound. At steady state, filaments would be disassembling at a rate that just balances the rate of renucleation of filaments to replace them (with the rate of disassembly much slower than filament extension). A given filament could be anywhere in the disassembly process, but the average filament bound at steady state will have lost half of its length. The average filament at steady state will thus occupy one-quarter of the RecA binding sites on a given DNA molecule. If 6.3% of the available DNA binding sites are occupied by RecA thus distributed in filaments that coat one-quarter of a DNA molecule, then we can estimate that 25.2% of the DNA molecules have some bound RecA protein. About 6% of the DNAs ([0.252]^2^, or approximately 0.06) will have two filaments bound. Half of these will have two filaments with the same orientation, and thus effectively have only one end where net disassembly will occur. The remaining 3% of DNAs with the potential for two bound filaments and two disassembling ends represent the maximum percentage of DNAs in this condition, and this maximum can only be approached late in the disassembly process after the DNA initially bound by oriented filaments has been vacated completely. Since the potential for multiple filaments is limited early in the dissociation curve, and 3% in any case is well within the error limits of our measurements, we have not adjusted our model or corrected our reported rates for this effect. To the extent that multiple disassembling ends contribute to the observed rates, they would lead to a very slight overestimate of the disassembly rate.

**Figure 9:**
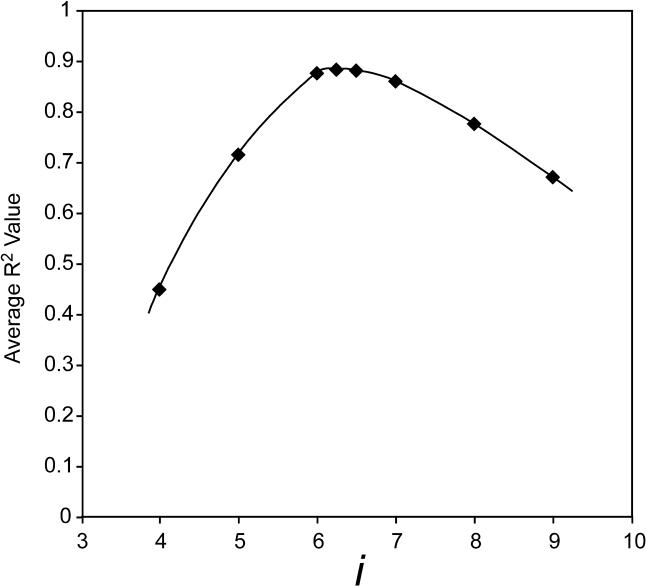
Quality of the Data Fitting, as Measured by *R* ^2^, Varies with Changes in *i* The calculation is illustrated for the 3-kbp DNA substrate. A maximum is seen at *i* = 6.4 monomers. For each of the 12 independent datasets used in this work, the measured *k*_cat_ was multiplied by the indicated value of *i* to get a predicted *k*_off_. This value of *k*_off_ was then used to fit the data to equation 1, constraining *k*_off_ and *k*_cat_ and allowing *k*_nuc_ to vary. *R* ^2^ is then a measure of the quality of the resulting fit. The *R* ^2^ value in each case is averaged for 12 trials.

The interval between waves of ATP hydrolysis, *i*, is *k*_off_ /*k*_cat_. We calculate *i* to be 6.0 ± 0.5, 6.2 ± 1.1, and 5.8 ± 1.1 monomers for the 2-, 3-, and 4-kbp DNA substrates, respectively. As with the other parameters, these values agree well and are identical within experimental error. When averaged across all 38 trials, *i* is 6.0 ± 0.9 monomers if *k*_cat_ is constrained and 6.0 ± 0.8 monomers if *k*_cat_ is not constrained (see Table 1). As noted in the Introduction, the quantitative disassembly model predicts a different *k*_off_ value for different values of *i*. To explore further the significance of this determination, we carried out an additional exercise. Each filament disassembly dataset was fit to equation 1 with *k*_cat_ held constant to the value measured before net filament disassembly and *k*_off_ held constant to the value predicted by the model for values of *i* from 4 to 9. When the quality of the data fit (as measured by *R* ^2^) is plotted against *i*, an optimum is seen at *i* = 6.4 monomers for the 3-kbp DNA substrate (Figure 9). The optimum is seen at *i* = 5.9 monomers for the 2-kbp DNA substrate, and at *i* = 5.8 monomers for the 4 kbp-DNA substrate (unpublished data).

## Discussion

**Figure 10:**
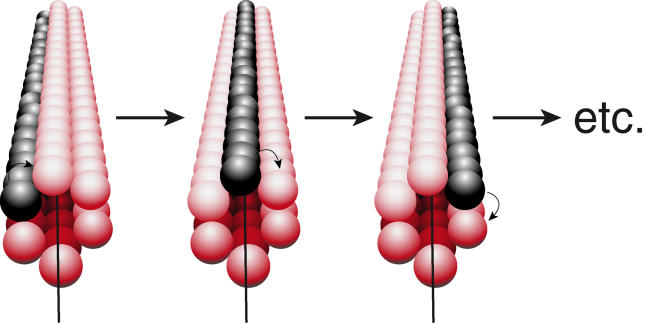
Coordination of ATP Hydrolytic Waves in a RecA Filament The dark monomers are those in the hydrolytic step of their hydrolytic cycle. The successive steps are separated by 0.5 s. The model shown assumes exactly six RecA monomers per helical filament turn and *i* = 6.0. As noted in the text, both the helical organization in the filament and the organization of ATP hydrolytic waves in RecA filaments may deviate slightly from this ideal. Note that there is no evidence that a RecA monomer in a filament is in contact with another RecA located six monomers away, so each of the gray balls shown in these illustrations are separated from their gray neighbors. An animated version of this model is available in Video S1.

We conclude that when RecA protein is bound to dsDNA, ATP hydrolysis within the filament occurs in highly organized and unidirectional waves. Successive waves occur at intervals of approximately six monomers and travel through the filament at a rate of approximately 120 monomers per minute. More precisely, the rate of RecA protein filament disassembly from dsDNA (which represents the ultimate wave) is 123 ± 16 RecA monomers per minute per filament end, under the conditions of these experiments. While this is occurring, ATP is being hydrolyzed by monomers within the filament with a measured *k*_cat_ of 20.8 ± 1.5 min^−1^. The ratio *k*_off_/*k*_cat_ gives *i*, the interval between the waves of hydrolysis within the filament. The *k*_off_/*k*_cat_ = *i* = 6.0 ± 0.9 monomers. These results are taken from data in which the *k*_cat_ for ATP hydrolysis was constrained to the value measured prior to the pH shift. The results are quite similar when *k*_cat_ is not so constrained (Table 1). Given that there are six RecA monomers per helical turn of a RecA filament, this relationship reveals a striking pattern of ATP hydrolysis by RecA protein, one in which the ATP hydrolytic events at a given moment are lined up along one longitudinal face of the filament (Figure 10). These “stripes” of ATP hydrolysis proceed around the circumference of the filament in six steps, with successive steps occurring at 0.5-s intervals. The overall pattern corresponds well to a rotary motor model for the coupling of RecA-mediated ATP hydrolysis to DNA strand exchange, and fulfills the last of three major predictions of that model [10]. The motor function of RecA may play a role in certain aspects of the repair of stalled replication forks [10].

The rate of filament disassembly that we measure is consistent with a rough estimate of 2.4 monomers per second (144 min^−1^), obtained by Libchaber and colleagues [34]. This earlier estimate was based on single molecule experiments using very long DNAs, and ADP buildup that can contribute to aggregate filament dissociation [54,55,70] was not controlled. Importantly, the rate of RecA filament disassembly from dsDNA is nearly twice that from ssDNA [27], reflecting the change in functional state that is observed when a second strand of DNA is introduced into a RecA filament [10,48,49]. The observed rate of filament disassembly on dsDNA is three times that predicted for a similar filament with no ATP hydrolytic coupling between adjacent monomers. At a minimum, this must reflect an elaborate coordination of ATP hydrolytic cycles for adjacent RecA monomers in the filament. The functional state of RecA protein filaments on dsDNA is, in every experimental sense tested to date, identical to the functional state observed during active DNA strand exchange [10,12,49,62]. We therefore propose that the current results directly reflect the status of RecA filaments that are actively promoting DNA strand exchange.

The extent of coupling between RecA monomers inferred from the current experiments can be correlated to information derived from EM observations of the nucleoprotein filament. The datasets fit best to equation 1 with a *k*_off_ corresponding to ATP hydrolysis reflecting *i* = 5.8–6.4 RecA monomers. The directly measured average *k*_off_/*k*_cat_ ratio for all datasets suggests that *i* = 6.0 ± 0.9 RecA monomers. Interestingly, previous EM reconstructions of the RecA-dsDNA complex indicate that there are 6.2 RecA monomers per helical turn of the nucleoprotein filament under at least some conditions [52]. This may imply an organization that is highly ordered, albeit not quite as ideal as the organization depicted in Figure 10.

The RecA crystal structure does not reveal any contact between every sixth monomer in the helix [71,72]. This suggests that coordination within the filament is mediated through adjacent monomer-monomer contacts. With ATP hydrolytic waves spaced at six-monomer intervals, it is tempting to postulate a hydrolytic cycle with six distinct steps. In one turn of the helix, there could be six slightly different monomer conformations, corresponding to various stages of binding, hydrolyzing, and releasing nucleotide. Indeed, multiple conformations of RecA protein within the RecA nucleoprotein filaments have been observed [73]. Unlike a hexameric circle (such as the hexameric helicases to which RecA appears related), the synchronization between steps in the hydrolytic cycle could readily produce a noninteger separation of ATP hydrolytic waves (e.g., 6.2 monomers) in a filament. Although our average value of 6.0 for the parameter *i* is consistent with the idealized picture of Figure 10, the experimental error could readily accommodate such noninteger outcomes.

The ATP hydrolytic cycle should now become the focus of more intensive investigation. We have little information about the steps in the cycle as they occur on RecA protein, nor do we know to which step dissociation of a RecA monomer might be coupled. For example, the dissociating form of RecA could be almost any species one could imagine as an intermediate in the ATP hydrolytic cycle, such as a RecA-ADP complex, a RecA-Pi complex, or any of a number of other possibilities. The model presented in Figures 3B and10 works regardless of the precise mechanism of ATP hydrolysis proposed.

The patterns of ATP hydrolysis we observe in RecA filaments are quite distinct from those documented for other major filament types. The tubulins [1,2,3] and the actins [4,5,6] are filament-forming proteins that bind and hydrolyze GTP and ATP, respectively. The NTP hydrolysis affects the capacity of the filaments to assemble or dissociate. The proteins most readily assemble as the ATP- or GTP-bound forms. Very slow hydrolysis of the bound NTP results in a form that is more readily dissociated from the filament [4,5,6]. These filaments thus expand and contract in a pattern that is dictated by NTP hydrolysis, as well as by the activities of numerous regulatory proteins. The assembly and disassembly produces work at the filament ends. The ADP- or GDP-bound monomers in the filament interiors do not exchange nucleotides to rebind ATP or GTP, so there is no active nucleotide turnover within the filaments. In contrast, ATP hydrolysis occurs uniformly throughout a RecA protein filament formed on DNA. This ATP hydrolysis can result in filament disassembly at one end, as we continue to document here. However, the steady-state hydrolysis of ATP in the interior of RecA filaments does not result in dissociation and has the capacity to do a different kind of work. The rotary motor illustrated in Figure 10 can be coupled to DNA strand exchange. That coupling is inferred by the capacity of RecA to drive strand exchange through heterologous DNA inserts [41,42], promote unidirectional strand exchange [13,43], and promote strand exchange between two duplex DNAs [42,44,45]—only when it is hydrolyzing ATP. The coupling is also seen in the predictable relationship that exists between the rates of ATP hydrolysis and branch movement during strand exchange [59], and in a kind of indirect DNA helicase reaction that RecA promotes with certain branched DNA substrates [74]. At least one potential biological role for this motor function can be found in the regression of stalled replication forks that is sometimes required for their repair [10,12,75,76,77]. Fork regression is promoted by RecA protein, but again only if ATP is hydrolyzed [10,78,79]. If RecA protein is bound to a chromosome at a replication fork, and ATP is available, the surrounding DNA will not remain static.

We do not expect the organization revealed here for ATP hydrolysis in RecA filaments to apply generally to the eukaryotic homologs of RecA. The eukaryotic Rad51 protein hydrolyzes ATP at rates 30- to 40-fold below those reported for bacterial RecA proteins. RecA protein generally requires ATP hydrolysis for extensive strand exchange [17,42,43], while Rad51 protein does not [80,81]. There are no reports that Rad51 can promote the kinds of reactions to which RecA protein couples ATP hydrolysis (four-strand exchange and strand exchange past a significant heterology in the DNA substrates) [82,83]. Also unlike RecA, the Rad51 protein promotes DNA strand exchange with no intrinsic polarity [82,84,85]. RecA-mediated ATP hydrolysis is thus likely to have a role unique to bacterial DNA metabolism or a role that is supplanted by other proteins in eukaryotes.

## Materials and Methods

### 

#### Proteins and biochemicals

E. coli RecA protein was purified to homogeneity as described [86]. Two different preparations were used for analysis, with one preparation having the final fraction subjected to an additional step. The protein was loaded onto a PBE-94 column equilibrated with R buffer (20 mM Tris-HCl [80% cation, pH 7.5], 1 mM dithiothreitol, 0.1 mM EDTA, and 10% [w/v] glycerol), and the column was developed with a linear gradient from 0 to 1.0 M KCl. The RecA protein was eluted at approximately 600 mM KCl, dialyzed extensively against R buffer, and concentrated as described. Results with both RecA protein preparations were the same and were combined.

E. coli SSB was purified as described [42,87]. The RecA protein and SSB concentrations were determined by absorbance at 280 nm, using extinction coefficients of ɛ_280_ = 0.59 A_280_ mg^−1^ ml [88] and ɛ_280_ = 1.5 A_280_ mg^−1^ml [89], respectively. RecA protein and SSB preparations were free of detectable endo- and exonuclease activities on dsDNA or ssDNA. Unless otherwise noted, all reagents were purchased from Fisher (Pittsburgh, Pennsylvania, United States). MES buffer, PEP, pyruvate kinase, lactate dehydrogenase, phosphocreatine, ATP, and NADH were purchased from Sigma (St. Louis, Missouri, United States). Creatine kinase and ATPγS were purchased from Roche Molecular Biochemicals (Indianapolis, Indiana, United States). Restriction enzymes were purchased from New England Biolabs (Beverly, Massachusetts, United States). T4 DNA polymerase was purchased from New England Biolabs, and T4 DNA ligase was purchased from Promega (Madison, Wisconsin, United States) and New England Biolabs. Dithiothreitol was purchased from Research Organics (Cleveland, Ohio, United States). Sephacryl S-500 and PBE-94 resins were purchased from Amersham Pharmacia Biotech (Piscataway, New Jersey, United States).

#### DNA substrates

Oligonucleotides were purchased from Integrated DNA Technologies. The concentrations of duplex DNA stock solutions were determined by absorbance at 260 nm using 50 μg ml^−1^ A_260_^−1^ as a conversion factor. All DNA concentrations are given in terms of total nucleotides unless otherwise noted.

The 3-kbp linear double-stranded DNA substrate with a 30-nt single-stranded 5′ tail was generated by first digesting pUC119 plasmid DNA [90] with two restriction enzymes, SapI and SmaI. After digestion, residual protein was removed by sequential 1:1 extractions with phenol/chloroform/isoamyl alcohol (25:24:1) and chloroform/isoamyl alcohol (24:1). The resulting 2,883- and 279-bp fragments were concentrated by precipitation in ethanol. The 2,883-bp fragment was separated from the 279-bp fragment using size-exclusion chromatography with Sephacryl S-500 resin and concentrated with an Amicon Microcon concentrator (Millipore, Billerica, Massachusetts, United States). Residual protein was removed by sequential 1:1 extractions with phenol/chloroform/isoamyl alcohol (25:24:1) and chloroform/isoamyl alcohol (24:1). The 2,883-bp fragment was concentrated by precipitation in ethanol. Two complementary oligonucleotides, 47 and 20 nt in length, were annealed and ligated in excess to the staggered end of the 2,883-bp fragment. Excess oligonucleotides were separated from substrate DNA using size-exclusion chromatography with Sephacryl S-500 resin. The substrate DNA was concentrated with an Amicon Microcon concentrator (Millipore). Residual protein was removed by sequential 1:1 extractions with phenol/chloroform/isoamyl alcohol (25:24:1) and chloroform/isoamyl alcohol (24:1). The substrate DNA was concentrated by precipitation in ethanol.

The 2-kbp linear dsDNA substrate with a 30-nt single-stranded 5′ tail was generated by first digesting pUC119 with EcoRI and AatII. The larger fragment was isolated via agarose gel extraction. The DNA ends were filled in with T4 DNA polymerase and ligated with T4 DNA ligase to result in the 2,223-bp pEAW373 plasmid DNA. The 4-kbp linear dsDNA substrate with a 30-nt single-stranded 5′ tail was generated by first digesting pUC119 with BanII that cuts at two sites, and the 2,766-bp fragment was separated from the 396-bp fragment via agarose gel extraction. Next, pACYC184 plasmid DNA [91,92] was digested with HindIII and AvaI, with the resulting 1,396-bp fragment isolated via agarose gel extraction. The DNA ends of the 2,766- and 1,396-bp fragments were filled in with T4 DNA polymerase and ligated with T4 DNA ligase to result in the 4,162-bp pEAW374 plasmid DNA. Both pEAW373 and pEAW374 maintained unique SapI and SmaI sites; they were treated in the same manner as the 3-kbp DNA substrate to yield 2- and 4-kbp linear dsDNA substrates with single-stranded 5′ tails. The lengths and sequences of the 5′ tails were the same in each of 2-, 3-, and 4-kbp DNA substrates.

To confirm the identity of the linear dsDNA substrates, a sample of each DNA substrate was digested with AflIII to yield a 133-bp fragment with a 4-nt 3′ tail and 30-nt 5′ tail. Digest of the duplex fragments without annealed oligonucleotides would have resulted in a 113-bp fragment with a 4-nt 3′ tail and 3-nt 5′ tail. Product separation in a 3.5% agarose gel confirmed the identity of the linear dsDNA substrate with a 30-nt 5′ tail and allowed quantitation of yield. In all DNA preparations used in these trials, the final yield of complete substrate DNA was greater than 97%.

#### Reaction conditions

All reactions were carried out at 37 °C in 25 mM buffer (detailed below), 10 mM magnesium acetate, 5% (v/v) glycerol, 1 mM dithiothreitol, 3 mM potassium glutamate, 3 mM ATP, an ATP regenerating system (10 units/ml pyruvate kinase and 3 mM or 2 mM PEP), and concentrations of DNA and RecA protein as described below and in figure legends. The coupled spectrophotometric assay also contained 10 units/ml lactate dehydrogenase and 3 mM NADH. Specific buffers used are described below and in the figure legends. DNA and protein concentrations are indicated for each experiment. Reactions were incubated for 10 min before ATP was added to start the reaction.

#### ATP hydrolysis assays**

A coupled spectrophotometric assay was used to measure DNA-dependent ATP hydrolysis by the RecA protein [29,93]. The regeneration of ATP from ADP and PEP was coupled to the oxidation of NADH and monitored by the decrease in absorbance of NADH at 380 nm. The 380-nm wavelength was used instead of the absorption maximum at 340 nm so that the signal would remain within the linear range of the spectrophotometer for the duration of the experiment. The assay was carried out using a Varian Cary 300 (Varian, Palo Alto, California, United States) dual beam spectrophotometer equipped with a temperature controller and 12-position cell changer. The cell path length and band pass were 0.5 cm and 2 nm, respectively. The NADH extinction coefficient at 380 nm of 1.21 mM^−1^ cm^−1^ was used to calculate rates of ATP hydrolysis.

#### Filament disassembly reactions

ATP hydrolysis reactions were started as described above in 25 mM MES/NaOH (76% anion, pH 6.62) buffer. Reactions contained 12 μM RecA protein, 12 μM (total nucleotides) linear duplex DNA, and the ATP and ATP regeneration conditions indicated above. These were incubated for approximately 20 min to reach a stable steady-state rate of ATP hydrolysis. The rate was determined and reflected a virtually complete binding of all available DNA by RecA protein. Under these conditions, we routinely achieved rates reflecting an apparent *k*_cat_ for bound RecA protein (one monomer per three available DNA base pairs) of 18–24 min^−1^. The few reactions displaying initial rates reflecting a *k*_cat_ less than 18 min^−1^ generally reflected a problem with one or another reagent, and were discarded. After preincubation to achieve a DNA substrate saturated with RecA protein, the solution was subjected to a pH shift. The 200 μl reaction was diluted with gentle mixing into 200 μl of a solution (also preincubated at 37 °C), containing 25 mM Tris-acetate (30% cation, pH 8.50) buffer, 10 mM magnesium acetate, 5% (v/v) glycerol, 1 mM dithiothreitol, 3 mM potassium glutamate, 3 mM ATP, an ATP regenerating system (10 units/ml pyruvate kinase and 3 mM or 2 mM PEP), and 0.3 μM SSB. The final pH of the reaction after the pH shift was 8.00. The RecA and DNA concentrations were halved as a result of the pH shift, but the concentrations of ATP, Mg^2+^, and ATP regeneration system components were kept constant. Following the pH shift, the production of ADP was monitored spectrophotometrically as the RecA filaments disassembled. The rate of ATP hydrolysis declined, eventually settling to a slow steady-state rate of ATP hydrolysis that reflects the minimal amount of bound RecA protein for a given experiment (see Results).

## Supporting Information

Video S1 Coordinated Waves of ATP Hydrolysis—The Movie This movie illustrates the model of Figure 10. The red RecA monomers are the ones hydrolyzing ATP at any given moment. The transitions from one set of monomers to the next occur every 0.5 s. In this animation, the 5′-proximal end of the initiating DNA strand (the strand that orders the orientation of the filament; see text) is at the upper left. The filament structure is based on the RecA structure of Story and Steitz [72], as cited in the text. (PDF not available).
(148 KB MOV).

## Abbreviations

ATPγS: adenosine 5′-*O*-(3-thiotriphosphate)
bp: basepair(s)
dATP: 3′-deoxyadenosine triphosphate
dsDNA: double-stranded DNA
EM: electron microscopy
kbp: kilobasepair(s)
nt: nucleotide(s)
NTP: nucleoside triphosphate
PEP: phosphoenolpyruvate
SSB: single-stranded DNA-binding protein of Escherichia coli
ssDNA: single-stranded DNA
